# New Maximum Likelihood Estimators for Eukaryotic Intron Evolution

**DOI:** 10.1371/journal.pcbi.0010079

**Published:** 2005-12-30

**Authors:** Hung D Nguyen, Maki Yoshihama, Naoya Kenmochi

**Affiliations:** Frontier Science Research Center, University of Miyazaki, Kiyotake, Miyazaki, Japan; University of California San Diego, United States of America

## Abstract

The evolution of spliceosomal introns remains poorly understood. Although many approaches have been used to infer intron evolution from the patterns of intron position conservation, the results to date have been contradictory. In this paper, we address the problem using a novel maximum likelihood method, which allows estimation of the frequency of intron insertion target sites, together with the rates of intron gain and loss. We analyzed the pattern of 10,044 introns (7,221 intron positions) in the conserved regions of 684 sets of orthologs from seven eukaryotes. We determined that there is an average of one target site per 11.86 base pairs (bp) (95% confidence interval, 9.27 to 14.39 bp). In addition, our results showed that: (i) overall intron gains are ~25% greater than intron losses, although specific patterns vary with time and lineage; (ii) parallel gains account for ~18.5% of shared intron positions; and (iii) reacquisition following loss accounts for ~0.5% of all intron positions. Our results should assist in resolving the long-standing problem of inferring the evolution of spliceosomal introns.

## Synopsis

When did spliceosomal introns originate, and what is their role? These questions are the central subject of the introns-early versus introns-late debate. Inference of intron evolution from the pattern of intron position conservation is vital for resolving this debate. So far, different methods of two approaches, maximum parsimony (MP) and maximum likelihood (ML), have been developed, but the results are contradictory. The differences between previous ML results are due predominantly to differing assumptions concerning the frequency of target sites for intron insertion. This paper describes a new ML method that treats this frequency as a parameter requiring optimization. Using the pattern of intron position in conserved regions of 684 clusters of gene orthologs from seven eukaryotes, the authors found that, on average, there is one target site per ~12 base pairs. The results of intron evolution inferred using this optimal frequency are more definitive than previous ML results. Since the ML method is preferred to the MP one for large datasets, the current results should be the most reliable ones to date. The results show that during the course of evolution there have been slightly more intron gains than losses, and thus they favor introns-late. These results should shed new light on our understanding of intron evolution.

## Introduction

Twenty-eight years have passed since the discovery of spliceosomal introns [1], but their evolution remains poorly understood. In the ongoing debate on intron evolution, the central issues include: introns-early versus introns-late, the mini-gene hypothesis, the proto-splice site hypothesis, rates of intron gain and loss, and ratio of parallel gain. Reconstruction of intron evolution from observed data is an important step toward resolution of these issues.

Although introns have high mutation rates, making it difficult to trace lineages through sequence homology, their positions are well conserved [2,3]. Therefore, it is possible to reconstruct intron evolution by comparing intron positions among sets of orthologous genes from different species. There are five basic steps involved in this reconstruction: step 1, sequence genomes and annotate the genes; step 2, select sets of orthologous genes among species; step 3, align orthologous genes to produce an intron presence/absence matrix; step 4, reconstruct a phylogenetic tree of the species studied; and step 5, make inferences of intron evolution based on the intron matrix and the phylogenetic tree. Among these, step 5 is perhaps the most critical, since erroneous inferences will lead to misunderstandings of intron evolution. However, to date this step remains the least investigated.

Two main approaches, maximum parsimony (MP) and maximum likelihood (ML), have been applied to determine intron evolution from the pattern of intron position conservation. The MP approach, based on the assumption that intron gain and loss events occur rarely in evolution, infers the most parsimonious scenario (measured as a function of gains and losses). Conversely, the ML approach infers a scenario with the highest probability of producing the observed data, for a given model of intron evolution.

In a large-scale study of intron evolution, Rogozin et al. [4] applied an MP method to infer intron gains and losses along each branch of a phylogenetic tree of eight eukaryotes using 684 gene orthologs. Their results demonstrated that intron density at the crown (plant–animal) ancestor is relatively high (nearly one-third of the density found in humans). Gains outweighed losses in some lineages; in others, the opposite trend was observed. However, overall there were two gains per loss, suggesting a more important role for intron gain than loss during the course of eukaryotic evolution. In another large-scale study of 2,073 sets of orthologs from four fungal species, Nielsen et al. [5] used an MP method to infer intron gains and losses, then used a probabilistic model to correct the numbers. They found that intron gains are on a par with intron losses in the four species investigated.

Roy and Gilbert [6,7] applied an ML method to the above mentioned dataset of eight eukaryotes (excluding one species). Their results were somewhat surprising: the genes of the crown ancestor were rich in introns (about two-thirds the density found in humans), and many lineages exhibited a notable excess of intron losses over gains. The ratio between gains and losses in this study was 0.7, suggesting a general trend toward decreased intron density. In another study, Qiu et al. [8] applied a Bayesian network method, also based on the ML principle, to infer the evolution of introns in ten gene families containing a total of 677 sequences. Their results suggest that many of the intron positions shared across various species are the result of independent gains, and are not due to conservation of intron position.

The results of the two ML methods clearly contradict each other and also differ significantly from results of the MP methods. This may be because of the different assumptions they made about the number of target sites; a target site is defined here as a possible position for intron insertion in the multiple-sequence alignment of all species. The number of target sites thereby includes all observed sites, plus possible sites that had introns in the past or may have introns in the future. The method of Roy and Gilbert [6,7] assumes that parallel gains do not occur at all, which will happen only when the number of target sites is many orders of magnitude larger than the number of observed sites. In contrast, the method of Qui et al. [8] assumes that the number of target sites is the number of observed sites.

In this paper, we propose a new ML method, the underlying basis of which is the treatment of target site number as a parameter requiring optimization. A log-likelihood function was formulated to allow optimization of the number of target sites, and an expectation-maximization (EM) algorithm was developed to optimize the log-likelihood function. Our results paint a different picture from those provided by previous ML methods.

## Results

### Test between the Coelomata and Ecdysozoa Hypotheses

**Figure 1:**
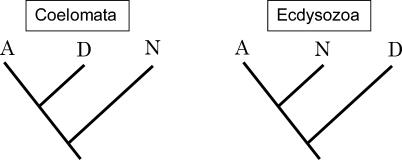
Two Hypotheses for the Relationship among Three Groups: Arthropods (A), Nematodes (N), and Deuterostomes (D) The coelomata hypothesis is shown on the left, and the ecdysozoa hypothesis is shown on the right.

**Figure 2:**
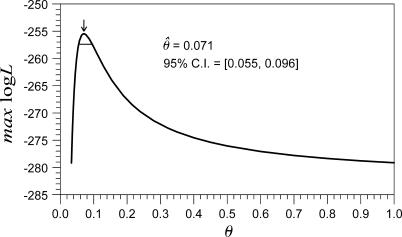
Relationship between *θ* and the Maximum Log-Likelihood Value The ecdysozoa phylogeny was used. The maximum log-likelihood value was calculated by treating all parameters other than *θ* as nuisance parameters and maximizing over them. The arrow shows the MLE of *θ* as 0.071. The horizontal line indicates a 95% CI of 0.055–0.096. The maximum log-likelihood value at the MLE of *θ* was −255.48.

**Figure 3:**
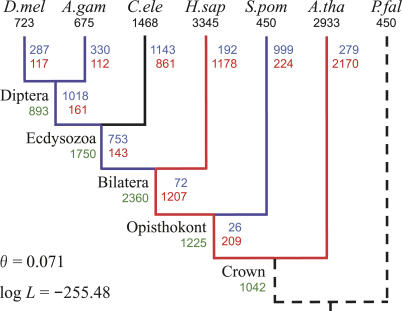
MLEs of the Number of Gains and Losses Using the Ecdysozoa Phylogeny Numbers of introns present in modern species (known) are in black. Numbers of introns present in ancestors (estimated) are in green. Numbers of gains and losses (estimated) are in red and blue, respectively. Branches that experienced >1.5 gains per loss are shown in red, and those that experienced >1.5 losses per gain are in blue. D.mel, *D. melanogaster;* A.gam, *A. gambiae;* C.ele, *C. elegans;* H.sap, *H. sapiens;* S.pom, *S. pombe;* A.tha, *A. thaliana;* P.fal, *P. falciparum*.

There are two hypotheses to explain the relationships of three eukaryotic groups: arthropods, nematodes, and deuterostomes (Figure 1) [9]. The coelomata hypothesis joins arthropods and deuterostomes, placing nematodes as the outgroup; the ecdysozoa hypothesis joins the arthropods and nematodes. Since our dataset includes representatives from all three groups (*Drosophila melanogaster* and *Anopheles gambiae* as arthropods, *Caenorhabditis elegans* as a nematode, and *Homo sapiens* as a deuterostome), we first tested both hypotheses using the pattern of intron position conservation. In our method, the maximum log-likelihood values for each tree configuration are computed, after which a χ^2^ test with one degree of freedom is performed for the value −2logΛ, where Λ is the likelihood ratio. The maximum log-likelihood value for the ecdysozoa hypothesis was −255.48 (Figures 2 and 3), whereas that for the coelomata hypothesis was −276.09 (Figure S1). Thus, −2logΛ = 41.22. Consequently, the coelomata hypothesis was rejected in favor of the ecdysozoa hypothesis (*p* < 10^−9^), a result consistent with that of another proposed method [9]. However, our method is simpler and appears to have a more sound mathematical basis. Hereafter, we will adopt the ecdysozoa hypothesis as the phylogenetic tree of the species studied.

### Reconstruction of Intron Evolution

The maximum likelihood estimator (MLE) of *θ* (see Materials and Methods for the definition of *θ*) was 0.071 (95% confidence interval [CI], 0.055–0.096; Figure 2). As a result, there was an average of one target site per 11.86 base pairs (bp) of sequence (95% CI, 9.27–14.39 bp), or 8.43 target sites per 100 bp (95% CI, 6.95–10.79).

Figure 3 shows the MLEs of the numbers of intron gains and losses along each branch of the phylogenetic tree. All branches leading to *H. sapiens,* and the terminal branch leading to *Arabidopsis thaliana,* experienced many more gains than losses. In contrast, all other branches except that leading to *C. elegans* experienced many more losses than gains. Losses along the terminal branch leading to *C. elegans* slightly outnumbered gains. In total, there were 6,382 gains versus 5,099 losses.

Figure 3 also shows the numbers of introns at internal nodes corresponding to the MLEs of the parameters. The intron density in the crown (plant–animal) ancestor was approximately one-third of that in *H. sapiens,* and increased until the bilateral ancestor appeared, after which it decreased in other lineages, while continuing to increase in *H. sapiens*. The intron density also increased in the terminal branch leading to *A. thaliana,* but decreased in the terminal branch leading to *Schizosaccharomyces pombe*. Although our method cannot infer the intron density at the root node of the phylogenetic tree, it inferred that 107 intron positions (the parameter *Q*_11_ in Protocol S1) were shared between *Plasmodium falciparum* and the crown ancestor. This number will become the lower bound for intron density at the root node if we apply the MP principle to the two deepest branches.

We also measured the frequencies of parallel intron gain and reacquisition following loss. Using our model of intron evolution, and incorporating the rates of intron gain and loss depicted in Figure 3, double parallel gains accounted for ~18.2% and triple parallel gains accounted for ~0.3% of the intron positions shared across two or more species (excluding *P. falciparum*). The probability that more than three introns would be gained in parallel at the same position was close to zero. Therefore, the total number of parallel intron gains accounted for ~18.5% of the shared intron positions. This result is slightly higher than the predicted range of 5% to 10%, but still less than the upper bound of 20% reported in a study using a different method on the same dataset [10]. The probability of reacquisition following loss was also small (~0.5% of the total intron positions).

The observed and expected (using the MLEs of the parameters) numbers of cases for each external intron pattern are presented in Table 1. Since many expected numbers are <5, the χ^2^ test cannot be used directly. Therefore, we grouped 75 intron patterns showing values of <3 into five new equally sized categories, and the result of the χ^2^ test was not statistically significant (χ^2^ = 36.15 with 56 df, *p* = 0.98).

**Table 1:**
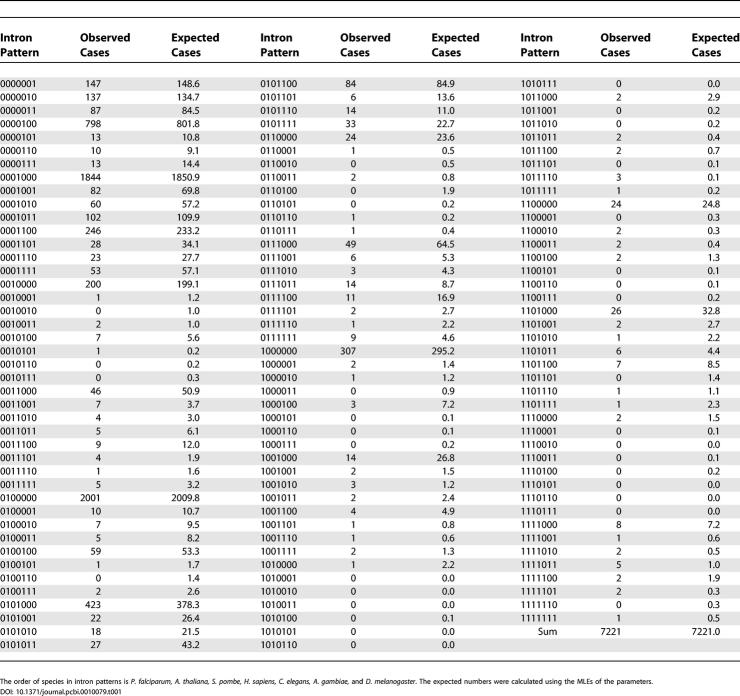
Observed and Expected Numbers of Cases for Each External Intron Pattern

### Verification of the Present Results

Since the log-likelihood function is highly complex and no closed form is available for calculating the MLEs of the parameters, three numerical methods were applied. The first was based on an EM algorithm, the second on a downhill simplex (DS) method, and the third on a genetic algorithm (GA). See the Materials and Methods section for details on all of these methods.

**Figure 4:**
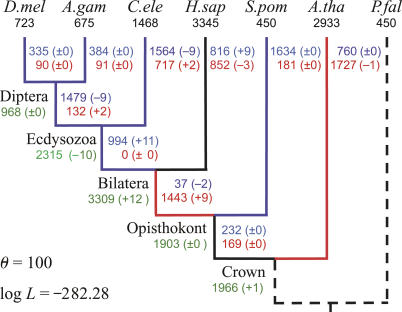
Comparison of Results between the EM algorithm (*θ* = 100) and a Previous ML Method [6] The results of the EM algorithm when using *θ* = 100 are shown. Numbers in parentheses indicate the differences between the two results using our results as the benchmark. Numbers of introns present in modern species (known) are in black. Numbers of introns present in ancestors (estimated) are in green. Numbers of gains and losses (estimated) for each branch are in red and blue, respectively. Branches that experienced >1.5 gains per loss are shown in red, and those that experienced >1.5 losses per gain are in blue. D.mel, *D. melanogaster;* A.gam, *A. gambiae;* C.ele, *C. elegans;* H.sap, *H. sapiens;* S.pom, *S. pombe;* A.tha, *A. thaliana;* P.fal, *P. falciparum*.

The present results were obtained using the EM algorithm, the advantage of which is its rapid convergence to a solution (Figure S2). However, its disadvantage is that its solution may not be the global maximum, but rather a local maximum, or even a stationary point. Therefore, the two other methods were used to verify whether or not the EM algorithm had found the global maximum. The DS method has the advantage that it does not require derivatives of the function to be optimized, and is less likely to be trapped in local maxima than other iterative methods such as EM. However, it is quite computationally expensive. The GA method is also unlikely to be trapped in local maxima, although its solution quality is not as high, and it is time-consuming. Over ten runs, neither DS nor GA methods showed an improvement in results; further, the best of each of these ten runs produced similar results to the EM algorithm (unpublished data). Therefore, it is highly likely that the EM algorithm found the global maximum. The fact that the EM algorithm, using *θ* = 100, produced almost the same results as a previous ML method [7] provides further support for this conclusion (Figure 4).

## Discussion

### Implications of Target Site Frequency

To the best of our knowledge, ours is the first estimate of frequency of target sites, and can be accounted for by two hypotheses. The first is the exon theory of genes, which postulates that present-day exons are remnants of primordial mini-genes (~45–60 bp long) from the time of the last common ancestor of prokaryotes and eukaryotes [11]. The second is the proto-splice–site hypothesis, which predicts that introns are not inserted randomly, but only into proto-splice sites. It is proposed that proto-splice sites contain the sequence MAG|R [12] or MAG|Gt [13], where M is A or C, and R is A or G. Based on our results, if the former hypothesis is true, then the average length of mini-genes should be ~12 bp. On the other hand, if the latter hypothesis holds true, there should be ~8.4 proto-splice sites per 100 bp. The average length of 12 bp, however, appears to be too short for mini-genes, which are supposed to be 45–60 bp long. Moreover, it is unlikely that introns mostly lost during the archaea period were then reacquired by *H. sapiens* and *A. thaliana*. Therefore, we believe that the proto-splice site hypothesis is the more likely explanation for our results. However, the estimated frequency of the pattern MAG|R in the whole genomes of the six species we examined, excluding *A. gambiae,* is 2%–3% (unpublished data) and therefore too small to explain the optimal value of 8.4%. Consequently, the consensus pattern of proto-splice sites may need to be re-determined.

### Relative Importance of Intron Gains and Losses

Our results show a slight excess (~25%) of intron gains over losses (6,382 versus 5,099). However, there was an excess of intron-lost branches over intron-gained branches (five versus four). Since these results may change upon addition of more species, the conclusions about the relative importance of intron gain and intron loss will depend upon the balance of future datasets. Assuming that the seven species studied here represent a balanced dataset, our conclusion is that intron gain has played a slightly larger role than intron loss during the course of eukaryotic evolution, but that the relative importance may vary with time and lineage.

Note that the results shown here were determined using the intron patterns in conserved alignment regions. We predict that the relative importance of intron gain and loss would tip more toward intron gain if complete alignments were used, as suggested by the results of Rogozin et al. [4]. However, we do not know what the effect of misalignments would be in this case.

Figure 3 shows that rates of intron gain and loss vary significantly among distantly related species, but only slightly among more closely related species (e.g., between *D. melanogaster* and *A. gambiae*). Moreover, except for the ecdysozoa–*C. elegans* branch in which intron gains and losses are approximately in balance, the other branches experienced either many more gains than losses, or losses than gains. There seems to be an inverse relationship between rates of gain and loss on each branch. One possible explanation for this pattern is that the rates of intron gain and loss may be controlled by selective pressure exerted through genome compaction and expansion. This pressure should vary significantly among distantly related species, but only slightly among those more closely related.

### Implications for the Introns-Early versus Introns-Late Debate

Two theories, introns-early and introns-late, have been proposed for the origin of spliceosomal introns. The introns-early theory asserts that introns were required to facilitate the assembly of early genes and were already present in the last common ancestor of prokaryotes and eukaryotes. Thus, this theory suggests that intron loss is the main driving force for intron evolution [14–17]. In contrast, the introns-late theory suggests that introns were gained after the emergence of eukaryotes and that intron gain plays a major role in the modern distribution of introns [18–20]. Many attempts have been made to resolve the debate (e.g., [21,22]). Although our results do not provide a definitive answer, they appear to lend more support for the introns-late theory. In particular, our results indicate that eukaryotic evolution has not been characterized by a general decrease in intron density, as predicted by the introns-early theory. With regard to the lineage leading to *D. melanogaster,* intron gains were dominant during the period from the crown ancestor to the bilateral ancestor, and intron losses were dominant after this point. There are two scenarios for intron evolution during the period between the last common ancestor of eukaryotes and the crown ancestor: either an increase or a decrease in intron density. As there would be only one turning point in the former, but two in the latter, the parsimony principle favors the former scenario in which intron density increased during the earliest stage of eukaryotic evolution.

### Comparison with Previous ML Methods

Our results differed greatly from those obtained by Roy and Gilbert [6,7], whose estimates for the numbers of introns at internal nodes were larger than ours. They determined an intron density in the crown (plant–animal) ancestor that was nearly twice that of our results. Their method also tended to infer fewer intron gains and more losses for each branch of the tree. On two branches, one from the crown ancestor to the opisthokont ancestor, and the other from the bilateral ancestor of *H. sapiens,* Roy and Gilbert's method predicted that gains and losses were approximately in balance. In contrast, our method predicted a notable excess of intron gains over losses. A possible explanation for these differences is that their model assumes that parallel intron gains do not occur [6,7]. This would lead to the assumption that the number of target sites must be extremely large, and would in turn bias the results. To test this hypothesis, we ran the EM algorithm with *θ* set to 100 (Figure 4), and the results generated were almost identical to those of Roy and Gilbert [6,7], thereby supporting our explanation for the differences. However, a valid value for *θ* should be in the range (0, 1), where *θ* = 0 indicates that the number of target sites is equal to the number of observed splice sites, and *θ* = 1 indicates that the number of target sites is equal to the total length of the alignments. Therefore, the value *θ* = 100 is unrealistic, since it implies that the number of target sites is approximately 100 times the total length of the alignments. Consequently, their results appear to underestimate intron gains and to overestimate losses.

Our results also contradicted the findings of Qui et al. [8], who determined that most intron positions shared between kingdoms were the result of parallel gains. Although different datasets were used, the contradiction may have occurred because their approach includes the assumption that the number of target sites equals the number of observed intron positions (i.e., equivalent to *θ* = 0 in our method). This assumption may lead to an overestimation of the rate of intron gain, which in turn leads to an overestimation of parallel gains. When we used *θ* = 0, our method produced results in which the ratio of parallel gain reached ~98.4%, thus supporting our explanation of the differences between the two methods. Additionally, since their dataset contains an average of 68 sequences per gene family, the total number of intron patterns is 2^68^. This is much larger than 49, which is the average number of intron positions per gene family. Thus, it is possible that their sample dataset was insufficiently large for a valid statistical inference.

Note that assumptions about the target site frequency of both previous ML methods represented extreme versions of our method (*θ* = 100 and *θ* = 0) and were outside the 95% CI (0.055–0.096). Their log-likelihood values were −282.28 and −2199.16, respectively. Thus, they were both rejected in favor of the MLE of *θ* (*p* < 10^−10^ by χ^2^ tests, 1 df).

### Comparison with MP Methods

Rogozin et al. [4] were the first group to apply MP to the problem of inferring intron gains and losses in the dataset that we used. However, they reported results only for the coelomata phylogeny; another group reported results for the ecdysozoa phylogeny [6,7]. When compared with our method, their method appears to underestimate the number of introns at all of the internal nodes, as well as the number of intron losses on all branches. Furthermore, the number of intron gains was underestimated on all internal branches, but overestimated on several terminal branches (e.g., those leading to *H. sapiens, D. melanogaster,* and *A. gambiae*). These variations are believed to be caused by the differences between ML and MP. In an MP method, the most parsimonious scenario is always chosen, even though the next-most parsimonious scenario has a close probability of occurrence. Let us consider an intron pattern in which the chances of the most and the next-most parsimonious scenarios occurring are 51% and 49%, respectively. In the MP method, 100% of cases will be assigned to the most parsimonious scenario, while none will be assigned to the next-most parsimonious. In the ML method, 51% and 49% of cases will be assigned to the most parsimonious and the next-most parsimonious scenarios, respectively.

Although the results obtained by the method of Nielsen et al. [5] are not available for the dataset used in this study, we believe that they should fall somewhere between the results of Rogozin et al. [4] and ours. Nielsen et al. [5] used a probabilistic model to correct some intron patterns, for which the possibility of the next-most parsimonious scenario was high, thereby reducing the gap between the results using ML and MP.

Although the debate between ML and MP is vigorous [23,24], much of it relates to the problem of phylogenetic tree reconstruction, and may not be relevant to the problem of intron evolution prediction. Errors in predicting the number of events can still lead to the correct tree. Therefore, we believe that ML will be a better predictor of intron evolution when there is a large amount of sample data, such the dataset used in this study. MP may be the method of choice when the sample dataset is insufficiently large for a valid statistical inference (such as the dataset of Qui et al. [8]). Investigating the performance of these two approaches in their prediction of intron evolution will be the subject of future work.

### Future Applications

The method presented here may also be applied to at least two other problems: (i) determination of the phylogenetic tree of eukaryotic species based on the conservation of intron positions; and (ii) inference of rates of gene gain and loss on a genomic scale. For the former, we would consider the phylogenetic tree *T* as a parameter needing to be optimized, and find the MLE of *T* over all tree configurations; for the latter, we would need to construct a gene presence/absence matrix, rather than an intron presence/absence matrix.

## Materials and Methods

### Dataset.

We used the 684 eukaryotic clusters of orthologous genes (KOGs), which are available at ftp://ftp.ncbi.nlm.nih.gov/pub/koonin/intron_evolution [4]. Each KOG contains genes from eight eukaryotes: *D. melanogaster, A. gambiae, C. elegans, H. sapiens, S. pombe, Saccharomyces cerevisiae, A. thaliana,* and *P. falciparum*. Multiple protein sequence alignments and intron presence/absence matrices of these KOGs were downloaded from the above site. Following Roy and Gilbert [6,7], we only extracted intron patterns in conserved alignment regions and excluded *S. cerevisiae* due to its sparse intron distribution. The total length of conserved alignment regions in the dataset was 488,157 bp. The number of intron positions in these conserved regions was 7,221; the number shared across two or more species was 1,787. When the outgroup *P. falciparum* was excluded, the number of intron positions was 7,049 and the number shared was 1,722.

### The log-likelihood function.

Our model of intron evolution assumes that introns are inserted independently and uniformly at all available target sites. Similarly, intron loss occurs with equal probability at all sites possessing introns, regardless of when they were inserted. Rates of gain and loss are assumed to be constant along each branch of the phylogenetic tree, but they can vary between branches. As suggested by the results of Rogozin et al. [4] and Stoltzfus et al. [25], our model does not allow “intron sliding.” However, our model does differ from some of the others in that it allows for both parallel gains and reacquisition following loss.

Given the model of intron evolution described above, our problem can be defined as follows: Let *N* be the number of species, *G* be the total length of all alignments in bp, and *T* be the phylogenetic tree of these species. Since the phylogenetic tree *T* is binary, it contains *N* external nodes, each corresponding to a species, *M* = *N −* 1 internal nodes, with each node corresponding to a divergent event, and *B* = 2*M* branches (including *N* external branches). An external intron pattern is a binary sequence *s*_1_ *s*_2_...*s_N_* of length *N*. When *s_i_* = 0 or 1 (*i* = 1, 2, ..., *N*), this means that an intron is absent or present at the *i*th external node. The definition of an internal intron pattern is similar, but with length *M* instead of *N*. There are 2*^N^* (indexed from 0 to 2*^N^ −* 1) possible external intron patterns, and 2*^M^* (indexed from 0 to 2*^M^ −* 1) internal intron patterns. For the *i*th external/internal intron pattern, the binary code of *i* will give the intron states of the external/internal nodes. For example, for *N* = 4, the binary code of external intron pattern *i* = 3 will be “0011.” Thus, an intron will be absent at the first and second species, yet present at the third and fourth species in this pattern. We denote *n_i_* (*i* = 0, 1, …, 2*^N^* − 1) as the number of cases for the *i*th external pattern, and *g_k_* and *l_k_* (*k* = 1, 2, ..., *B*) as the number of gains and losses along the *k*th branch. We then have to estimate *g_k_* and *l_k_* for a given set of *n_i_*. Note that *n*_0_ (i.e., the number of target sites without introns) cannot be observed, and must be treated as a parameter requiring optimization.

Denoting *S* and *P* as the numbers of observed splice sites (intron positions) and target sites in the dataset, respectively, we have:


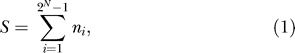



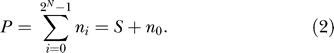


It follows from Equation 2 that *n*_0_ = *P* − *S*. Since *P* must ≤*G* (i.e., the total length of all alignments), *n*_0_ must be constrained by: 0 ≤ *n*_0_ ≤ *G* − *S*. If we define 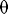 such that: 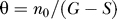 , then from Equation 2 we get: 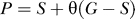 .

We denote 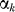 and 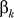 (*k* = 1, 2, …, *B*) as the probabilities of changing state from 0 to 1 (intron gain) and from 1 to 0 (intron loss) along the *k*th branch, respectively; *b_k_* and *e_k_* (*k* = 1, 2, …, *B*) as the node indexes of the beginning and end nodes of the *k*th branch, respectively; and *s_h_* (*h* = 1, 2, …, *N* + *M*) as the intron state of the *h*th node. The expected number of cases for the complete intron pattern *ij* (*i* = 0, 1, …, 2*^N^* − 1, *j* = 0, 1, …, 2*^M^* − 1), which is the combination of the *i*th external pattern and the *j*th internal pattern, is calculated by:


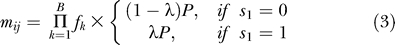


where:


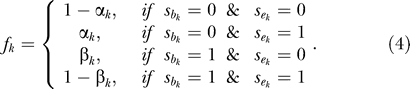


Here 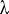 is a variable denoting the fraction of target sites having introns at the root node and *s*_1_ is the intron state of the root node.

Then the probability *p_i_* (*i* = 0, 1, …, 2*^N^* − 1) for the *i*th external pattern is:


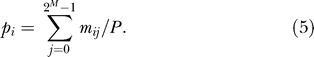


Finally, the likelihood function is:


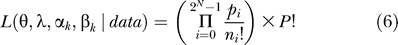


and the log-likelihood function is:


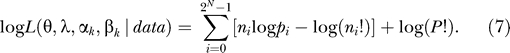


We have to find the MLEs 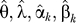 that maximize the log-likelihood function. Note that with our definitions, the values of 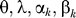 are all in the (0, 1) interval. We will denote 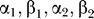 as the probabilities of intron gain and loss along the two deepest branches, respectively. Then we have the following two propositions:

Proposition 1: There are infinite sets of MLEs 
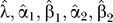
 .

Proof: See Protocol S1.

Proposition 1 indicates that the number of introns at the root node, as well as the numbers of gains and losses along the two deepest branches, cannot be determined without additional information. Therefore, these values will not be reported in the Results section.

Proposition 2: There are 2*^N^* ^−2^ sets of MLEs 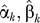 (*k* = 3, 4, …, *B*).

Proof: See Protocol S2.

We define the most biologically meaningful solution in these 2*^N^* ^−2^ sets of MLEs 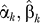 (*k* = 3, 4, …, *B*) as the one having the least sum of variances for intron gains and losses among all branches, i.e., having the smallest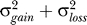 .

### The EM algorithm.

An EM algorithm was proposed for calculating the MLEs of 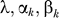 , given a fixed value of 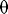 . The Brent algorithm was used for finding the MLE of 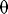 based on the profile likelihood method, i.e., by treating all other parameters as nuisance parameters and maximizing over them. Implementation of the Brent algorithm is problem-independent and straightforward: we simply applied the code in the book *Numerical Recipes in C* [26]. However, implementation of the EM algorithm is problem-specific. In general, EM is an iterative algorithm comprising two steps: the E-step and the M-step [27]. Our implementation of these two steps is as follows:

E-Step: In our problem, the complete dataset comprises the number of cases for all complete intron patterns (i.e., including external and internal nodes). The conditional expected number of cases for the complete intron pattern *ij* (*i* = 0, 1, …, 2*^N^* − 1, *j* = 0, 1, …, 2*^M^* − 1) given *n_i_* is calculated as follows:


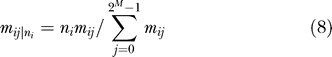


where *m_ij_* is calculated based on Equation 3 using the current set of parameters 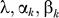 .

M-Step: In this step we must find a new set of parameters that maximize the likelihood of the complete data conditioned by the observed data. First, we calculate the conditional expected numbers of gains and losses for each branch *k* of the phylogenetic tree by:


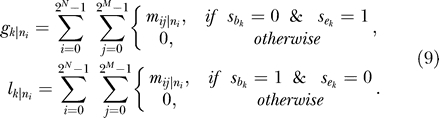


Next, the conditional expected number of introns at each node *h* is calculated by:


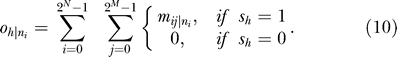


Finally, the new parameters are calculated by the following formulas:


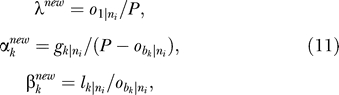


where *o*_1|*ni*_ is the conditional expected number of introns at the root node.

First, we set the value of λ to *S*/2*P*, so that the introns are present in half of the observed intron positions at the root node; then set the values of 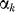 to 0.01 and the values of 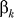 to 0.1 (*k* = 1, 2, …, *B*). The two steps described above are then repeated until the difference 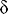 between the new set and the current set of parameters, which is calculated based on Equation 12, is smaller than a predefined value (10^−8^ in our algorithm).


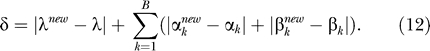


The source code of the EM algorithm was written in C language and is available on request to the corresponding author.

### The DS method.

To implement the DS method, we used the C code in [26] with only two minor modifications: the first limits all parameters to optimization within the (0, 1) range; and the second repeats the main procedure 50 times, in order to obtain a higher-quality solution.

### The GA method.

Our GA is based on a multi-population steady-state GA [28] and can be run in parallel on a cluster of PCs to obtain results more quickly**.** In this GA, only one offspring solution is produced, either from two parental solutions by crossover, or from one parental solution by mutation in each generation. The offspring is immediately inserted into the population, and if it is fitter it replaces the worse parent. Linear ranking selection with a bias of 1.25 was used for selecting the parents. The population size was set to 400 individuals, which were divided equally into eight subpopulations. The mutation rate was set to 0.5.

## Supporting Information

Figure S1 MLEs of the Numbers of Gains and Losses Using the Coelomata Phylogeny (11 KB PDF)

Figure S2 Convergence of the EM Algorithm (*θ* = 0.071) (17 KB PDF)

Figure S3 General Form of an Internal Node (9 KB PDF)

Protocol S1 Proof for Proposition 1 (80 KB PDF)

Protocol S2 Proof for Proposition 2 (90 KB PDF)

## Abbreviations

bp: base pair
CI: confidence interval
DS: downhill simplex
EM: expectation-maximization
GA: genetic algorithm
KOG: eukaryotic cluster of orthologous genes
ML: maximum likelihood
MLE: maximum likelihood estimator
MP: maximum parsimony
