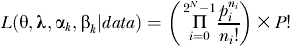# Correction: New Maximum Likelihood Estimators for Eukaryotic Intron Evolution

**DOI:** 10.1371/journal.pcbi.0020028

**Published:** 2005-12-30

**Authors:** Hung D Nguyen, Maki Yoshihama, Naoya Kenmochi

In *PLoS Computational Biology,* volume 1, issue:DOI: 10.1371/journal.pcbi.0010079

An *n_i_* was omitted from Equation 6. The correct equation is as shown below: