# Supplementary material for: New Maximum Likelihood Estimators for Eukaryotic Intron Evolution
Source: PLoS Comput Biol. 2005 Dec 30;1(7):e79. doi: 10.1371/journal.pcbi.0010079 (PMC1323467; doi:10.1371/journal.pcbi.0010079)
Supplement: Figure S1 — (11 KB PDF) [file pcbi.0010079.sg001.pdf]

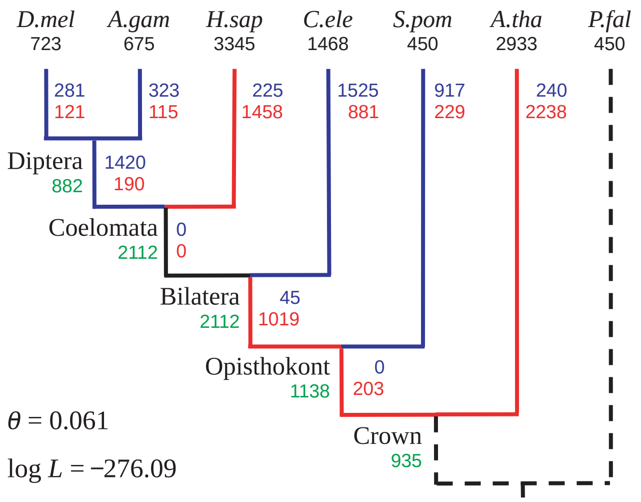

Figure S1. MLEs of the numbers of gains and losses using the coelomata phylogeny. Numbers of introns present in modern species (known) are in black. Numbers of introns present in ancestors (estimated) are in green. Numbers of gains and losses (estimated) are in red and blue, respectively. Branches that experienced >1.5 gains per loss (or losses per gain) are shown in red (or blue). D.mel, *D. melanogaster*; A.gam, *A. gambiae*; C.ele, *C. elegans*; H.sap, *H. sapiens*; S.pom, *S. pombe*; A.tha, *A. thaliana*; P.fal, *P. falciparum*.
