# Supplementary material for: New Maximum Likelihood Estimators for Eukaryotic Intron Evolution
Source: PLoS Comput Biol. 2005 Dec 30;1(7):e79. doi: 10.1371/journal.pcbi.0010079 (PMC1323467; doi:10.1371/journal.pcbi.0010079)
Supplement: Figure S2 — (17 KB PDF) [file pcbi.0010079.sg002.pdf]

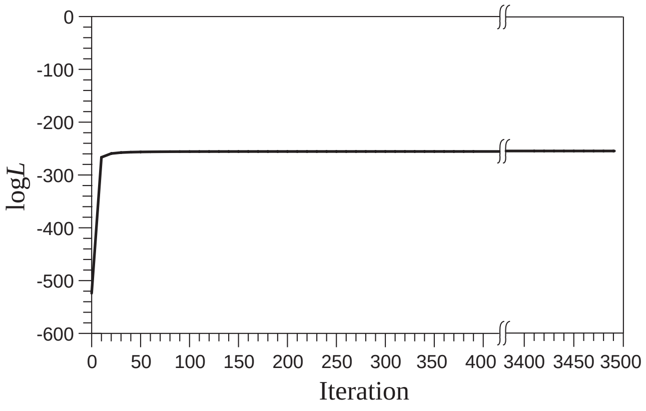

Figure S2. Convergence of the EM algorithm (θ = 0.071). The horizontal axis shows the number of iterations and the vertical axis shows the log-likelihood value. It can be seen that the EM algorithm quickly converged to the solution.
