# Supplementary material for: New Maximum Likelihood Estimators for Eukaryotic Intron Evolution
Source: PLoS Comput Biol. 2005 Dec 30;1(7):e79. doi: 10.1371/journal.pcbi.0010079 (PMC1323467; doi:10.1371/journal.pcbi.0010079)
Supplement: Figure S3 — (9 KB PDF) [file pcbi.0010079.sg003.pdf]

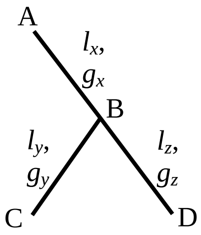

Figure S3. General form of an internal node. *B* is an internal node, *A* is its parent, and *C* and *D* are its children. l_x, g_x, l_y, g_y, l_z, and g_z are the numbers of intron losses and gains along branches *AB*, *BC*, and *BD*, respectively.
