# Supplementary material for: New Maximum Likelihood Estimators for Eukaryotic Intron Evolution
Source: PLoS Comput Biol. 2005 Dec 30;1(7):e79. doi: 10.1371/journal.pcbi.0010079 (PMC1323467; doi:10.1371/journal.pcbi.0010079)
Supplement: Protocol S1 — (80 KB PDF) [file pcbi.0010079.sd001.pdf]

Protocol S1: Proof for Proposition 1

Equations 3 and 5 can be rewritten as follows:

$$m_{iq} = \prod_{k=3}^{B} f_k \times \begin{cases} Q_{00} & if\ s_2 = 0\ \&\ s_3 = 0 \\ Q_{01} & if\ s_2 = 0\ \&\ s_3 = 1 \\ Q_{10} & if\ s_2 = 1\ \&\ s_3 = 0 \\ Q_{11} & if\ s_2 = 1\ \&\ s_3 = 1 \end{cases}, \qquad (13)$$

$$p_i = \sum_{q=0}^{2^{M-1}-1} m_{iq} \Big/ P, \qquad (14)$$

where:

$$Q_{00} = P(1-\lambda)(1-\alpha_1)(1-\alpha_2) + P\lambda\beta_1\beta_2, \qquad (15)$$

$$Q_{01} = P(1-\lambda)(1-\alpha_1)\alpha_2 + P\lambda\beta_1(1-\beta_2), \qquad (16)$$

$$Q_{10} = P(1-\lambda)\alpha_1(1-\alpha_2) + P\lambda(1-\beta_1)\beta_2, \qquad (17)$$

$$Q_{11} = P(1-\lambda)\alpha_1\alpha_2 + P\lambda(1-\beta_1)(1-\beta_2). \qquad (18)$$

Here, m_{iq} ($i = 0, 1, \ldots, 2^N - 1$, $q = 0, 1, \ldots, 2^{M-1} - 1$) is the expected number of cases for the intron pattern iq, which is the combination of the ith external pattern with the qth internal pattern excluding the root node. s_2 and s_3 are the intron states of the two nodes, which are children of the root node.

It is clear from equations 13 and 14 that p_i (and consequently the log-likelihood value) will be invariant if Q_{00}, Q_{01}, Q_{10}, Q_{11}, α_k, and β_k ($k = 3, 4, \ldots, B$) are constant. Moreover, the sum of Q_{00}, Q_{01}, Q_{10}, and Q_{11} is P, so there is one redundant equation in the four equations 15–18. As a result, there will be infinite sets of parameters λ, α_1, β_1, α_2, and β_2 that produce the same values for Q_{00}, Q_{01}, Q_{10}, and Q_{11} (since there are five variables but only three equations). It follows that there will be infinite sets of MLEs $\hat{\lambda}, \hat{\alpha}_1, \hat{\beta}_1, \hat{\alpha}_2, \hat{\beta}_2$.
