# Supplementary material for: New Maximum Likelihood Estimators for Eukaryotic Intron Evolution
Source: PLoS Comput Biol. 2005 Dec 30;1(7):e79. doi: 10.1371/journal.pcbi.0010079 (PMC1323467; doi:10.1371/journal.pcbi.0010079)
Supplement: Protocol S2 — (90 KB PDF) [file pcbi.0010079.sd002.pdf]

Protocol S2: Proof for Proposition 2

Any internal node B (except the root node) on the phylogenetic tree always has the form illustrated in Figure S3. Let A be the parent and C and D be the two children of B. Denote l_x, g_x, l_y, g_y, l_z, and g_z as the numbers of intron losses and gains along branches AB, BC, and BD, respectively. We will first prove that the log-likelihood value is invariant if we substitute l_x, g_x, l_y, g_y, l_z, and g_z with l'_x, g'_x, l'_y, g'_y, l'_z, and g'_z such that:

$$g'_x = P - o_A - g_x, \qquad l'_x = o_A - l_x,$$

$$g'_y = o_B - l_y, \qquad l'_y = P - o_B - g_y, \tag{19}$$

$$g'_z = o_B - l_z, \qquad l'_z = P - o_B - g_z.$$

Here o_A, o_B, o_C, and o_D are the numbers of introns at nodes A, B, C, and D, respectively, before the substitution.

Let o'_A, o'_B, o'_C, and o'_D be the numbers of introns at nodes A, B, C, and D, respectively, after the substitution. Since the number of introns at the root node and the probabilities α, β of all higher branches of A are invariant, the number of introns at node A is also invariant, that is:

$$o'_A = o_A. \tag{20}$$

From the definition of intron gains and losses we have:

$$o_B = o_A + g_x - l_x, \qquad o'_B = o'_A + g'_x - l'_x. \tag{21}$$

From equations 19, 20, and 21 we get:

$$o'_B = P - o_B. \tag{22}$$

Similarly, we can get:

$$o'_C = o_C, \qquad o'_D = o_D. \tag{23}$$

That is, the numbers of introns at nodes C and D are also invariant.

If we denote Φ by:

$$\Phi = \sum_{k=1, k \notin \{x,y,z\}}^{B} f_k \times \begin{cases} (1-\lambda)P, & if\ s_1 = 0 \\ \lambda P, & if\ s_1 = 1 \end{cases}, \tag{24}$$

then from equation 3 we can write:

$$\begin{aligned} & m_{ij|s_A=0,s_B=0,s_C=0,s_D=0} = \Phi(1-\alpha_x)(1-\alpha_y)(1-\alpha_z) \\ & = \Phi\left(1-\frac{g_x}{P-o_A}\right)\left(1-\frac{g_y}{P-o_B}\right)\left(1-\frac{g_z}{P-o_B}\right) \end{aligned}. \tag{25}$$

Substitute equations 19, 20, 22, and 23 to equation 25 we get:

$$\begin{aligned} & m_{ij|s_A=0,s_B=0,s_C=0,s_D=0} = \Phi\left(\frac{g'_x}{P-o'_A}\right)\left(\frac{l'_y}{o'_B}\right)\left(\frac{l'_z}{o'_B}\right). \\ & = \Phi\alpha'_x\beta'_y\beta'_z = m'_{ij|s_A=0,s_B=1,s_C=0,s_D=0} \end{aligned} \tag{26}$$

Using a similar method we get:

$$m_{ij|s_A=0,s_B=1,s_C=0,s_D=0} = m'_{ij|s_A=0,s_B=0,s_C=0,s_D=0}. \tag{27}$$

The same also holds true for all other intron patterns (s_A, s_C, s_D). Thus, in general:

$$m_{ij|s_B=0} = m'_{ij|s_B=1},\ \ m_{ij|s_B=1} = m'_{ij|s_B=0}. \tag{28}$$

Therefore, from equation 5 we get:

$$p'_i = \sum_{j=0}^{2^M-1} m'_{ij} \Big/ P = \sum_{j=0}^{2^M-1} m_{ij} \Big/ P = p_i. \tag{29}$$

Thus, p_i is invariant after the substitution. It follows from equation 7 that the log-likelihood value is also invariant. Since each internal node excluding the root node will have two sets of MLEs, there will be in total 2^{N-2} sets of MLEs $\hat{\alpha}_k, \hat{\beta}_k$ (k = 3, 4, …, B).
